# Tarter-Emetic in Pneumonia

**Published:** 1870-04

**Authors:** 


THE same author explains the tolerance of tartar-emetic in pneumonia, thus: When the pneumonia is extensive there is diminution of the respiratory surface, that is to say, the beginning of asphyxia, which is the cause of the tolerance, as we shall presently prove. In pneumonias of limited extent, however, and which only occasion a slight obstacle to respiration, the tolerance of tartar-emetic does not exist.

If in a case of pneumonia sufficiently extensive to diminish the respiratory surface, and to determine at the same time intense fever, the emetic does not provoke vomiting, it happens that, in consequence of the imperfect interchange of gas and atmospheric air in the lung, the carbonic acid is not eliminated from the blood; this accumulation of carbonic acid, which is characteristic of asphyxia from its beginning, paralyses certain portions of the nervous system, either of the periphery or of internal organs.

Amongst these latter nerves is the pneumo-gastric, in that portion of it which is distributed to the respiratory and gastric organs; hence the antimonial poison no longer produces upon the gastric filaments of this nerve, either at their periphery or at their centre, the centripital impression which is the point of departure of all vomiting. The vomiting is, in fact, nothing else than the reflex excitation of the nerves which are distributed to the diaphragm and to the abdominal muscles whose contraction effects the evacuation of the stomach. In this effort, the muscles of the abdominal cavity alone act, *the stomach is nearly inert*, it may on this account be replaced experimentally by a pig's bladder. This contraction of the muscles, and the nervous excitation in which it originates, can occur only by the aid of a central or peripheral impression; now, if the sensitive nerves have lost their sensibility, the first impression can no longer be produced, there is no more vomiting; it is exactly this impression which the carbonic acid destroys in the nerves.

Further, when carbonic acid exists in great quantity in the blood, there is perceptible not only a cutaneous anæsthesia, but likewise an anæsthesia of deep seated organs; the visceral nerves, especially the pneumo-gastric no longer respond to impressions, absolutely as the nerves of the skin. Tartarised antimony once injected into the veins, may in passing through the vascular system, excite the gastric termination of the vagus nerve, or its origin in the interior of the medulla, which thus provokes vomiting. Tickling of the throat occasions vomiting by peripheric excitation of the pneumo-gastric, the pharynx owing its sensibility in great part to a branch of the vagus nerve.

A CHAIR of anthropology has just been established at Florence in favor of professor Mantegazza, now professor of physiology at the University of Pavia.

